# Supplementary material for: Complexome profiling of the Chlamydomonas psb28 mutant reveals TEF5 as an early PSII assembly factor
Source: Plant Cell. 2025 Mar 17;37(6):koaf055. doi: 10.1093/plcell/koaf055 (PMC12164590; doi:10.1093/plcell/koaf055)
Supplement: koaf055_Supplementary_Data [file koaf055_supplementary_data.zip › psb28_interactive.zip!/plots/intensity/qualityPlots/peptides/VKE1_Cre12.g493150.t1.2.html]

### Cre12.g493150.t1.2

Trivial name: VKE1  
SeqCoverage [median]: 23.40  
Mass: 35.47 kDa  
  
 

| Sequence | MissedCleavage | Mass [Da] | wtIntensity | muIntensity | Score |
| --- | --- | --- | --- | --- | --- |
| AEAAAAAPEAVAVAP | 0 | 1307.67 | 3797.50 | 9854.23 | 112.75 |
| AFPTWVIGGK | 0 | 1074.59 | 2496.87 | 0.00 | 72.48 |
| AMDLAAR | 0 | 746.37 | 0.00 | 2414.92 | 110.61 |
| LNAVGAR | 0 | 699.40 | 7130.34 | 12855.63 | 181.15 |
| QAADSSAEGVAGR | 0 | 1217.56 | 1807.14 | 4373.23 | 189.57 |
| QAADSSAEGVAGRR | 1 | 1373.66 | 0.00 | 665.75 | 34.00 |
| SPVVATESSSR | 0 | 1118.56 | 10018.74 | 15189.55 | 200.71 |
| TIEGELLLDEVEKELAR | 1 | 1956.04 | 1954.99 | 0.00 | 64.65 |
|  |  |  | 27205.6 | 45353.3 |  |
